# Co-doped MnFe_2_O_4_ nanoparticles: magnetic anisotropy and interparticle interactions

**DOI:** 10.3762/bjnano.10.86

**Published:** 2019-04-12

**Authors:** Bagher Aslibeiki, Parviz Kameli, Hadi Salamati, Giorgio Concas, Maria Salvador Fernandez, Alessandro Talone, Giuseppe Muscas, Davide Peddis

**Affiliations:** 1 Department of Physics, University of Tabriz, Tabriz 51666-16471, Iran; 2 Department of Physics, Isfahan University of Technology, Isfahan, 84156-83111, Iran; 3 Dipartimento di Fisica, Università di Cagliari, S.P. Monserrato-Sestu km 0,700, 09042 Monserrato (CA), Italy; 4 Dipartimento di Scienze, Università degli Studi Roma Tre, via della vasca navale, 84 - 00146 Roma, Italy; 5 Department of Physics, University of Oviedo, Campus de Viesques, 33204 Gijón, Spain; 6 Istituto di Struttura della Materia-CNR, 00015 Monterotondo Scalo (RM), Italy; 7 Department of Physics and Astronomy, Uppsala University, Box 516, SE-751 20 Uppsala, Sweden; 8 Department of Chemistry and Industrial Chemistry (DCIC), University of Genova, Genova, Italy

**Keywords:** cobalt doping, collective dynamics, ferrite nanoparticles, interparticle interactions, magnetic properties

## Abstract

The effect of cobalt doping on the magnetic properties of Mn_1−_*_x_*Co*_x_*Fe_2_O_4_ nanoparticles was investigated. All samples consist of ensembles of nanoparticles with a spherical shape and average diameter of about 10 nm, showing small structural changes due to the substitution. Besides having the same morpho-structural properties, the effect of the chemical composition, i.e., the amount of Co doping, produces marked differences on the magnetic properties, especially on the magnetic anisotropy, with evident large changes in the coercive field. Moreover, Co substitution has a profound effect on the interparticle interactions, too. A dipolar-based interaction regime is detected for all samples; in addition, the intensity of the interactions shows a possible relation with the single particle anisotropy. Finally, the sample with the strongest interaction regime shows a superspin glass state confirmed by memory effect dynamics.

## Introduction

A strong scientific interest has driven the fundamental research on magnetic nanoparticles in the last decades [1–4], with interest constantly fed by their wide range of potential applications, e.g., from catalysis [5] and microwaves applications [6] to biomedicine, such as MRI [7], hyperthermia [8], and drug delivery [7,9] applications. Nanometer-sized magnetic materials exhibit different properties compared their bulk counterparts [10–11]. Below a critical radius, magnetic nanoparticles (NPs) organize themselves as a single magnetic domain, where all magnetic moments align in the same direction forming a “super spin” with a magnitude of 10^3^–10^4^ Bohr magnetons [12]. Due to the similarity of such superspins with atomic magnetic moments, even if with different characteristic time scale and much larger total moment, the magnetic behavior of mono-domain NP ensembles is often described as supermagnetism [12]. For non-interacting particles, above a so-called blocking temperature *T*_B_, their supermoments are thermally active in a corresponding time window, where they spontaneously reverse their direction in a superparamagnetic (SPM) regime, in analogy to atomic paramagnetism [10]. On the other hand, in concentrated ensembles of NPs, interparticle interactions can arise from long-range magnetostatic forces or local exchange coupling among particles [13–14]. Such interactions can be due to a strong influence on the overall magnetic behavior of the ensembles, inducing co-operative regimes showing super ferromagnetic (SFM) and superspin glass (SSG) behavior [13,15–16].

Among nanostructured materials, magnetic ferrite nanoparticles (Me^II^Fe_2_O_4_; Me^II^ = Fe^2+^, Ni^2+^, Co^2+^, Mn^2+^, etc.) are particularly appealing for technological applications thanks to their rich crystal chemistry, which can be altered in order to tune their magnetic properties [17–18]. They have a face-centered cubic (fcc) structure with cubic close-packed oxygen ions and tetrahedral (*T_d_*) and octahedral (*O_h_*) interstitial sites, which can be occupied by divalent and trivalent metal cations. By definition, the fraction of divalent ions in the octahedral sites represents the inversion degree (γ), which distinguishes them from the so-called “normal” structures where divalent and trivalent cations occupy the *T_d_* and *O_h_* sites, respectively. The cations within the same kind of interstitial site are ferromagnetically ordered due to oxygen-mediated superexchange interactions (J_Td–Td_ and J_Oh–Oh_). Additionally, the two sub-lattices are antiferromagnetically aligned, but with uncompensated moments, hence showing a final net magnetization (ferrimagnetism) which depends directly on the specific population of *T_d_* and *O_h_* sites. Furthermore, the magnetic anisotropy of the system is related to the specific cationic population and distribution in the different interstitial sites [19].

In the present paper, we investigate the structural and magnetic properties of ensembles of ferrite nanoparticles with formula Mn_1−_*_x_*Co*_x_*Fe_2_O_4_, (0 ≤ *x* ≤ 1) prepared by a combined low-energy ball milling and self-combustion method. This simple and low cost synthesis approach (i.e., the synthesis is performed at a relatively low temperature, not higher than 350 °C) allows nanoparticles to be produced with good control of size and crystallinity in large scale (i.e., tens of grams), much more than can be provided by conventional chemical approaches (usually limited to 100–300 mg). Therefore this method can be easily implemented for large-scale nanoparticle applications, such as for permanent magnets and biomedical applications.

Given their good particle size distribution (≈10 nm diameter) and almost the same saturation magnetization per particle, these samples represent a good model system to study the systematic effect of Co substitution on the magnetic properties of the whole ensemble. The Co substitution does not only affect the single particle anisotropy energy, and thus the intrinsic magnetic anisotropy of individual particles, but also the overall interacting regime among them. Because the samples are dense ensembles of particles in close proximity, special attention was devoted to the analysis of the interactions, showing the competitive effect of magnetic anisotropy and interparticle interactions. Finally, it is worth to noting that having particles with the same size and saturation magnetization but with different magnetic anisotropy opens interesting perspectives for applications in biomedical fields (e.g., MRI, drug delivery, hyperthermia) [20–21] and energy harvesting.

## Experimental

### Synthesis

Several samples consisting of manganese ferrite nanoparticles with different cobalt doping, i.e., Mn_1−_*_x_*Co*_x_*Fe_2_O_4_ (*x* = 0, 0.25, 0.5, 0.75, 1) were synthesized following a simple method based on solid-state ball milling and calcination of nitrate precursors and citric acid, discussed previously to prepare pure MnFe_2_O_4_ [22]. Manganese nitrate (Mn(NO_3_)_2_·4H_2_O, Merck, 99%), iron nitrate (Fe(NO_3_)_3_·9H_2_O, Merck, 99%), cobalt nitrate (Co(NO_3_)_2_·6H_2_O, Merck, 98.5%) and citric acid (C_6_H_6_O_7_, Merck, 99.5%) powders were mixed in a 1:1 molar ratio of total metal nitrates to citric acid. The powders were milled for 1 h in a planetary ball mill using agate balls, producing an amorphous precursor (Supporting Information File 1, Figure S1), which in a following step is annealed in air atmosphere at 350 °C for 3 h. This double step approach ensures good crystallization of small particles with relatively narrow size distribution. The samples were named Cn, where *n* = 0, 25, 50, 75, 100 is the percentage of cobalt.

### Experimental techniques and data treatments

X-ray diffraction patterns were collected using Cu Kα (λ = 0.154 nm) radiation with a Philips EXPERT MPD diffractometer. The average crystallite size was obtained from the Debye–Scherrer equation:

[1] $$\langle {\text{D}}_{\text{XRD}}\rangle =\frac{C\lambda }{w\cos\theta }$$

where *w* is the full-width at half-maximum (FWHM) of the XRD peaks, θ is the Bragg angle, *C* is the Scherrer constant related to the shape of crystallites (≈0.9 for spherical ones) and λ is the X-ray radiation wavelength. The lattice constant (*a*) of the samples was calculated using the following Bragg condition for cubic structures:

[2] $$\frac{1}{d^{2}}=\frac{h^{2}+k^{2}+l^{2}}{a^{2}}$$

where *d* is the interplanar distance and (*h*, *k*, *l*) are the Miller indices.

Transmission electron microscopy (TEM) analysis was performed with a JEM-2100 instrument using an accelerating voltage of 200 kV, with the nanoparticles deposited on a copper grid. The average particle size was obtained by measuring the diameter of more than 100 particles randomly selected in different parts of the grid.

DC magnetization measurements were carried out with a vibrating sample magnetometer (maximum field of 2 T) and a Quantum Design SQUID magnetometer, equipped with a superconducting coil that produces magnetic fields in the range from −5 T to +5 T. After the synthesis, the samples were in the form of a dry powder. About 5 mg of that powder was distributed inside a small transparent capsule (whose moment is absolutely negligible compared to that of the sample). A drop of epoxy resin was then deposited on top and allowed to dry overnight. The procedure allows the resin to diffuse around the sample preventing the physical rotation of the particles during the measurements. However, it is not able to solubilize individual particles, which continue to form large coarse aggregates, maintaining the original interparticle distance. The saturation magnetization *M*_S_ was extrapolated by fitting the *M*(*H*) curves at high field using the law of approach to saturation [23]:

[3] $$M=M_{\text{S}}\left(1-\frac{A}{H}-\frac{B}{H^{2}}\right)$$

where *A* and *B* are constant parameters.

Magnetization versus temperature measurements were performed using the zero-field-cooled (ZFC) and field-cooled (FC) protocols. The sample was cooled from room temperature to 5 K in a zero magnetic field; then a static magnetic field of 2.5 mT was applied. *M*_ZFC_ was measured during the warming up phase from 5 to 300 K, and *M*_FC_ was recorded during the subsequent cooling down from 300 to 5 K. The field-dependent isothermal remanent magnetization (IRM) and direct current demagnetization (DCD) were measured at 5 K. In the IRM measurement process, the demagnetized samples were cooled from 300 to 5 K in a zero magnetic field. Then a small external field was applied only for a few seconds, and the remanence was measured (*M*_IRM_). The process was repeated, increasing the field in progressive steps up to 5 T. In DCD measurements, the samples were cooled down to 5 K and then saturated by applying an external field of −5 T for a few seconds. Then, the remanent magnetization (*M*_DCD_) was measured such as in the IRM protocol increasing the field up to +5 T.

AC magnetic susceptibility measurements were performed by an AC susceptometer system. The measurements were carried out by cooling the sample from room temperature to 100 K in zero magnetic field, then magnetic susceptibility was measured during the warming up process in a magnetic field of 1 mT at frequencies of 33, 111, 333, 666 and 1000 Hz.

Mössbauer spectra were recorded at room temperature using a source of ^57^Co in Rh, in transmission geometry. The velocity scale was calibrated using a 25 μm Fe foil; the isomer shift values are referred to metal iron. The spectra were analysed as a superposition of two components with peaks of Lorentzian shape. The components were a sextet and a doublet, corresponding to the magnetically ordered and non-ordered components, respectively.

## Results and Discussion

**Figure 1:**
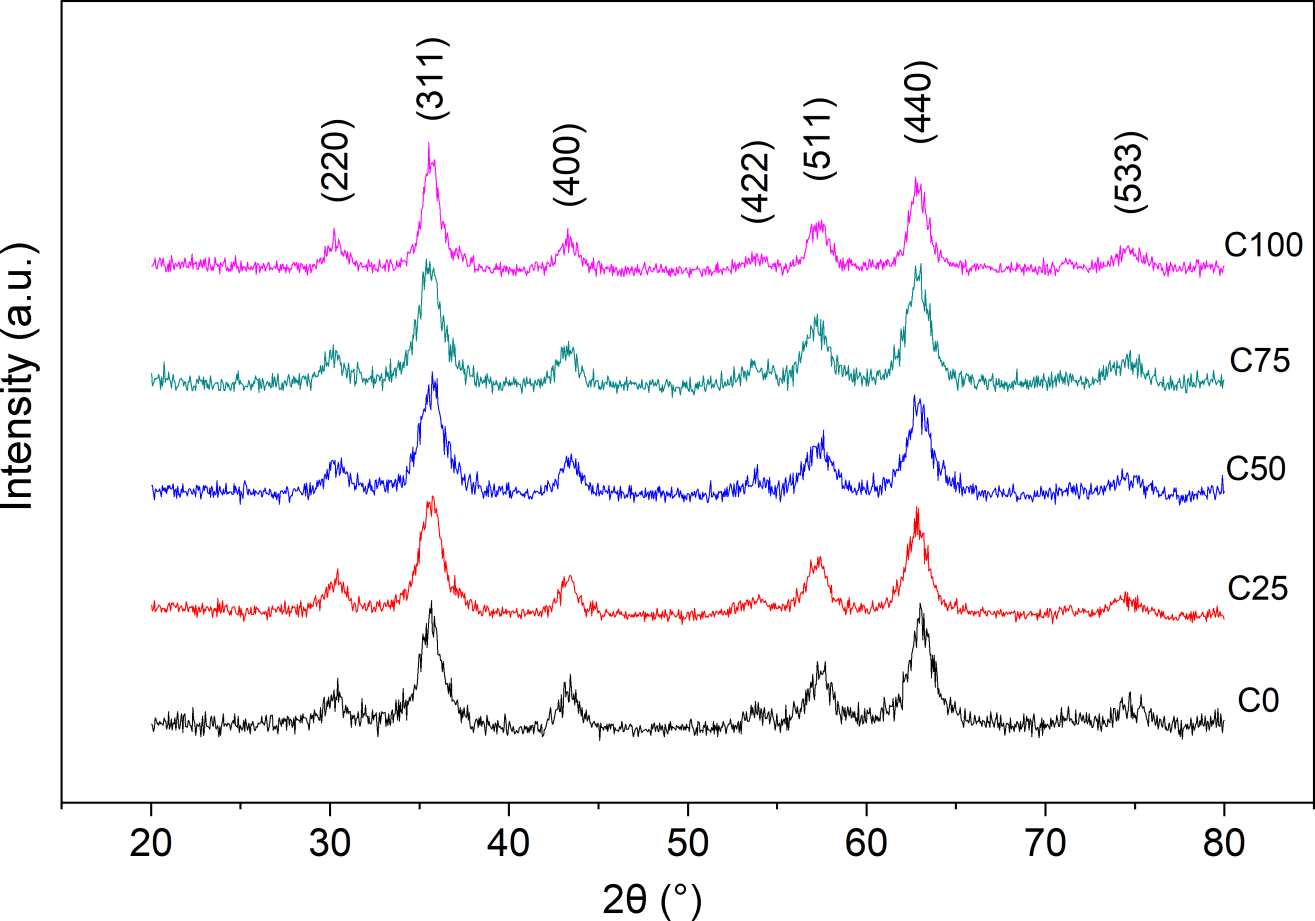
Room temperature X-ray diffraction patterns of all samples. All diffraction peaks are compatible with the spinel structure.

Figure 1 shows the room temperature X-ray diffraction patterns of the samples. They confirm a cubic spinel structure, comparable with those of single phase MnFe_2_O_4_ (PDF Card No. 73-1964) and CoFe_2_O_4_ (PDF Card No. 22-1086) with no impurity phases detected in any sample. The crystallite size is estimated by Scherrer’s formula to be between 7 and 8 nm for all the samples (Table 1). The lattice constants, calculated by Equation 2, are almost equal for all the samples, which indicate that Co doping does not induce any significant structural variation. However, the obtained values (about 8.34 Å) are smaller than those reported for bulk CoFe_2_O_4_ (8.38 Å) and MnFe_2_O_4_ (8.51 Å) [24]. This can be justified by the effect of the cationic distribution. In most cases, the cation distribution of stoichiometric bulk Mn-ferrite is demonstrated as


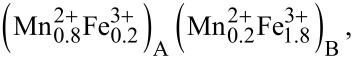


where A and B denote tetrahedral and octahedral sites in spinel structure [25]. A higher amount of Mn in B sites has been shown to reduce the lattice parameter to 8.4 Å in 7.5 nm MnFe_2_O_4_ nanoparticles [26]. At the nanoscale, the cation distribution of the spinel structure is deeply affected by the local broken bonds that lead to a coordination variation at the particle surface. Furthermore, the oxidation of Mn^2+^ to Mn^3+^ is a common event that accrues in the transition from the bulk state toward nanoscale, contributing to the effective cation distribution [26–27]. The difference in radius between Mn^2+^ (0.80 Å) and Mn^3+^ (0.66 Å) reduces the average lattice parameter. On the other hand, Co^2+^ randomly substitutes the two cations, but it has an intermediate radius of 0.74 Å, which on average does not affect the lattice parameter to a large extent, as experimentally observed in our samples.

**Table 1:** Average crystallite size 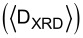 and lattice parameter (*a*). Uncertainties in the last digit are given in parentheses.

Sample	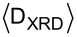 (nm)	*a* (Å)

C0	7.1(2)	8.33(4)
C25	7.5(3)	8.34(4)
C50	6.8(2)	8.35(3)
C75	7.2(3)	8.35(2)
C100	8.1(3)	8.34(4)

**Figure 2:**
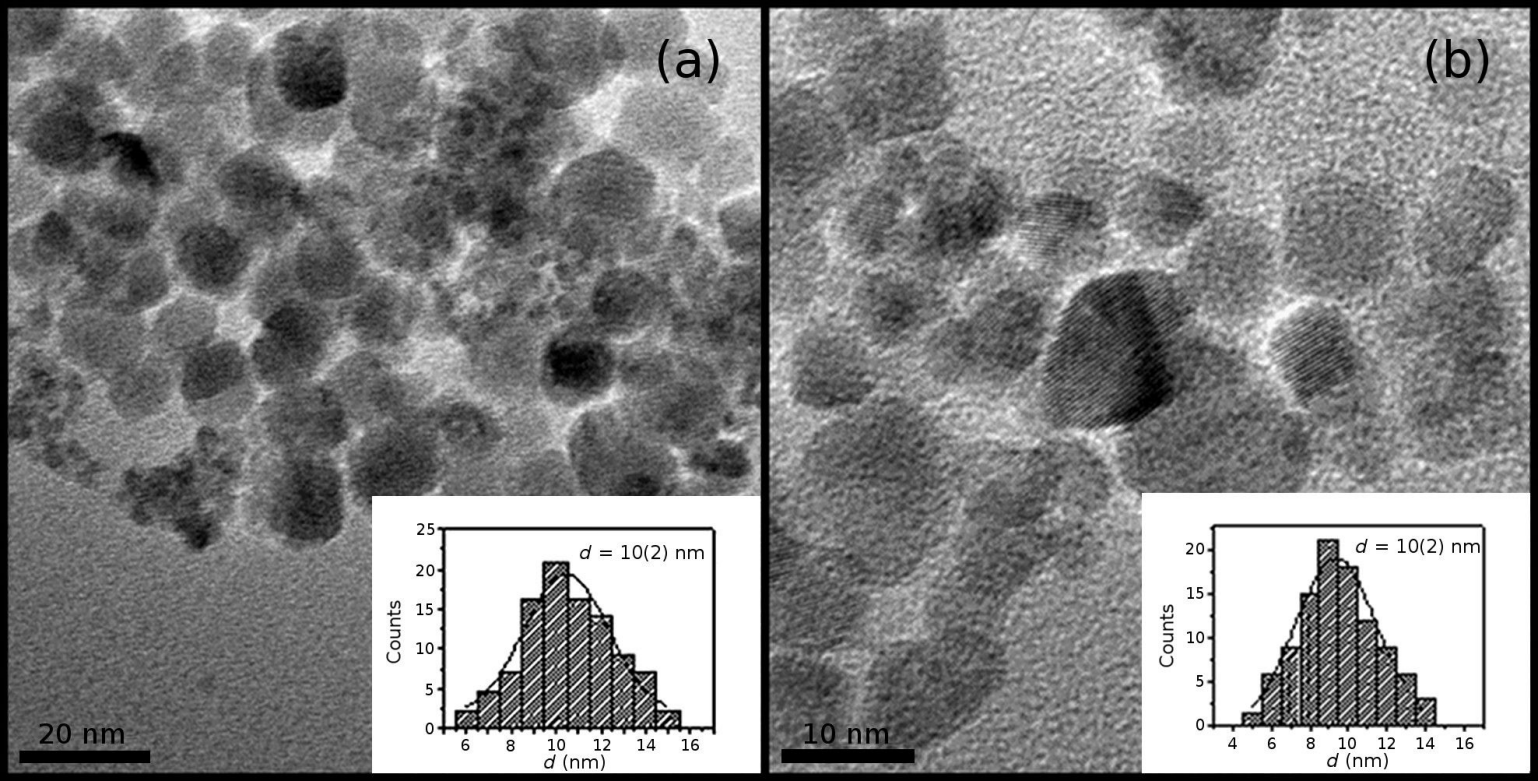
As an example of the size, shape and crystalline quality of the samples, TEM images of C0 (a) and C100 (b) samples are reported. The insets show the respective particle size distribution fitted with a Gaussian distribution (solid line).

TEM analysis shows a regular morphology of the NPs. All samples have spherical particles of uniform size distribution as in the examples provided in Figure 2 for C0 and C100 samples. Despite some aggregation, it is possible to measure the average particle diameter of 10.5(2) and 9.4(2) nm for C0 and C100 samples, respectively. Note that these dimensions are larger than the average crystallite size estimated from the XRD results, suggesting the presence of a disordered shell around the single-crystalline core.

### Magnetization dynamics

All of the Co-doped samples showed irreversibility in the FC and ZFC curves (Supporting Information File 1, Figure S2). It is well known that in ensembles of magnetic nanoparticles, the FC curve diverges from the ZFC curve, and the system shows magnetic irreversibility behaviour below a given temperature (*T*_irr_), which is related to the blocking of the biggest particles [28–29]. C0 is the only sample to show a maximum in the ZFC curve in the explored temperature range (5–300 K). Such a maximum is related to the temperature *T*_max_ = 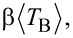 where 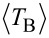 is the average blocking temperature and β is a constant (its value, β = 1–2, depends on the *T*_B_ distribution) [29–30]. Finally, the FC curves show an almost temperature-independent low-temperature trend, with even a low temperature negative slope for sample C0 below *T*_max_: this behaviour indicates the presence of interparticle interactions bringing the system in a collective state with higher anisotropy at low temperature [31–33]. For sample C0, where a strong interacting regime is expected, magnetization dynamics of superspin has been investigated. The in phase component (χ’) shows a peak at a temperature *T*_p_ that confirms the DC magnetization behavior. *T*_p_ shifts toward higher temperatures by increasing the applied frequency (Supporting Information File 1, Figure S3a). We tried to fit the frequency dependence of *T*_p_ with the Arrhenius law (Equation 4), the phenomenological Vogel–Fulcher law (Equation 5), and the power law (Equation 6):

[4] $$\tau =\tau_{\text{0}}\exp\left(\frac{E_{\text{a}}}{k_{\text{B}}T_{\text{p}}}\right)$$

[5] $$\tau =\tau_{\text{0}}\exp\left[\frac{E_{\text{a}}}{k_{\text{B}}\left(T_{\text{p}}-T_{\text{0}}\right)}\right]$$

**Figure 3:**
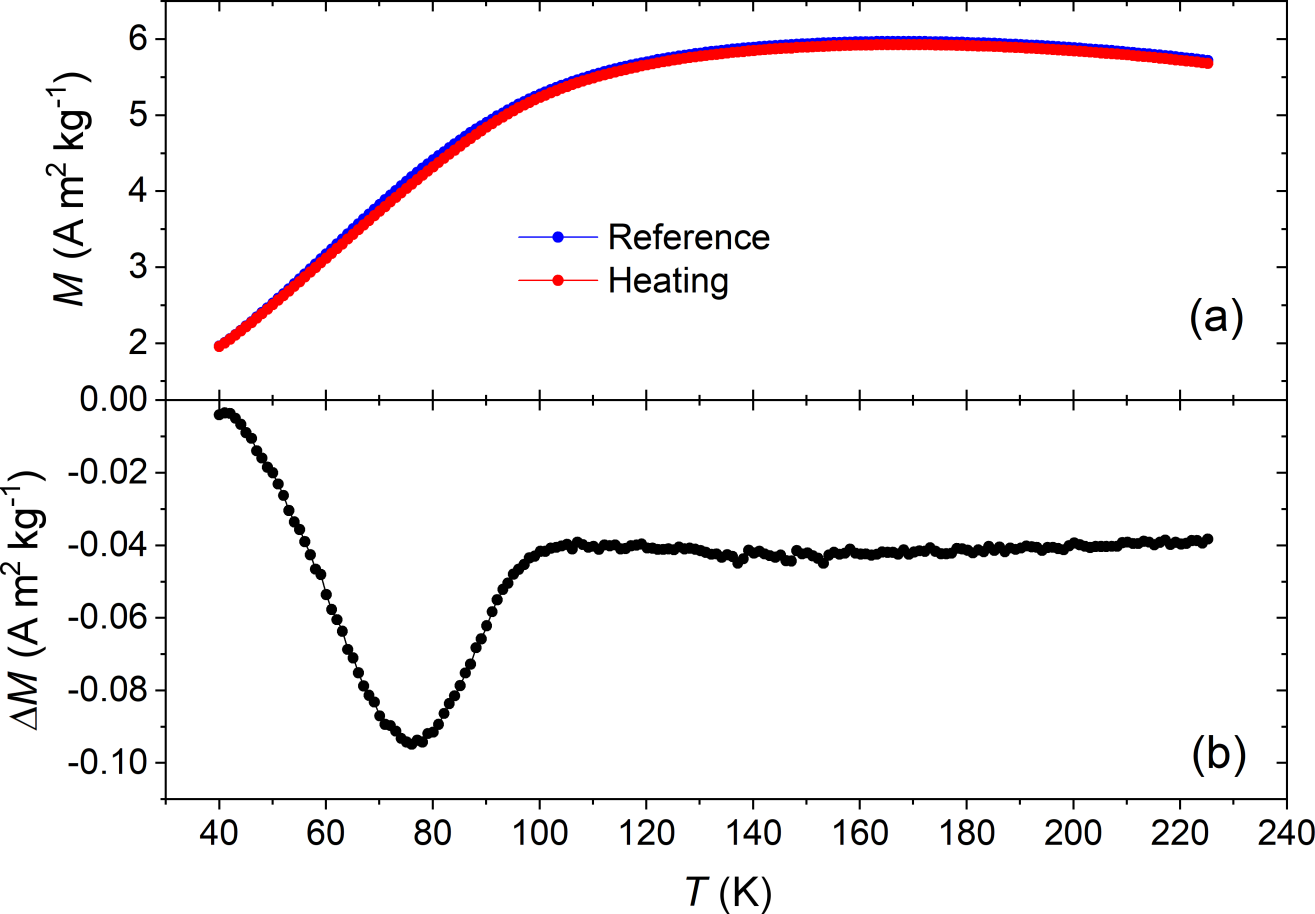
(a) Comparison of ZFC curve during cooling (blue circles) and subsequent warming up (red circles). The difference, Δ*M*, is reported in (b) as a function of temperature.

[6] $$\tau =\tau_{\text{0}}{\left(\frac{T_{\text{p}}}{T_{\text{g}}}-1\right)}^{-zv}$$

where *E*_a_ is the anisotropy energy of the single particle, *k*_B_ the Boltzmann constant and τ_0_ the characteristic relaxation time. The fit to the Arrhenius law led to unphysical values of the characteristic relaxation time and anisotropy constant. Since this model describes a non-interacting system, the results confirmed the presence of magnetic interactions between nanoparticles as reported in the literature for similar systems [34–36]. For interacting superparamagnetic particles, the phenomenological Vogel–Fulcher law gives a better description, introducing the value *T*_0_ as the temperature at which a collective behavior emerges [37–38]. When the interactions increase and superspin glass features characterize the collective state, the system can be better analyzed by the power law [13,39–40], as defined by Equation 6. Here the system exhibits a collective random freezing of moments below the glass temperature *T*_g_. Both the Vogel–Fulcher and power-law model fit our data well. Hence, to confirm possible spin glass dynamics, the investigation has been extended to non-equilibrium dynamics, i.e., memory effects. As a reference curve, a conventional ZFC magnetization vs temperature curve has been measured. Then the sample is cooled again in zero field, but held for 3 h at 80 K, thus below the hypothetical freezing temperature *T*_g_ ≈ 190 K, resulting from the power law fit. A clear decrease of magnetization is observed (Figure 3), which is a fingerprint of the superspin glass regime [41–42].

**Figure 4:**
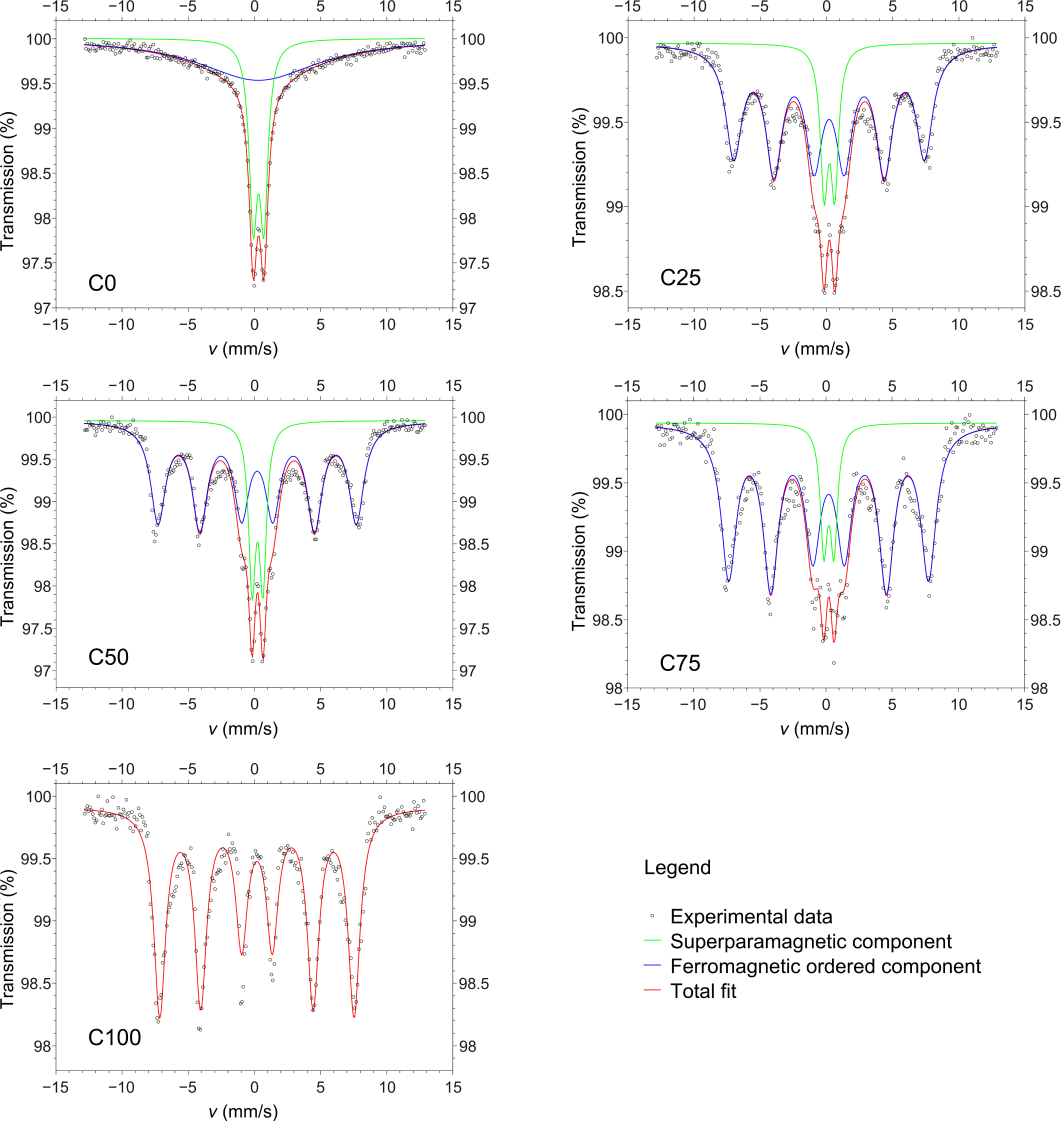
Mössbauer spectra recorded at 300 K for all samples. Only sample C0 is fully superparamagnetic at room temperature, while the only blocked sample is C100.

The samples were investigated by ^57^Fe Mössbauer spectroscopy at room temperature to estimate the superparamagnetic fraction of the sample at a given temperature. Figure 4 shows the spectra with the fit of the total signal and the subcomponents due to the ferromagnetic ordered (six lines) and superparamagnetic non-ordered (two lines) fractions; the results of the fits are shown in Table 2. All of the samples containing Co are partially magnetically ordered, with the exception of sample C100, which is totally ordered. The hyperfine magnetic field is constant from C25 to C100. The C0 sample shows the smallest magnetically ordered component (65%), which collapses into a single broad peak instead of a clear sextet. The superparamagnetic blocking temperature is directly proportional to the effective anisotropy energy of the particles [12]. From the Mössbauer data, it is clear that the Co substitution produces a general increment of the anisotropy. The Co^2+^ ions produced a marked magneto-crystalline contribution to the anisotropy in the spinel structure, more than Mn^2+^ and Mn^3+^ ions. Indeed, the crystal field does not entirely quench its orbital magnetic moment, allowing for a spin–orbital coupling responsible for the increased anisotropy, which is particularly strong for Co^2+^ ions located in octahedral sites [17,43–45]. However, it is interesting that 25% substitution with cobalt produces a large increment in C25 with respect to C0, while the subsequent increments induce only minor additional variations.

**Table 2:** Data extracted from the fit of the Mössbauer spectra. Isomer shift (IS), quadrupole splitting (QS), hyperfine magnetic field (*B*_hf_) and relative percentage area of the components. All measurements have an uncertainty of 1 in the last digit.

Sample	IS (mm/s)	QS (mm/s)	*B*_hf_ (T)	Area (%)

C0	0.41	–	–	65
	0.42	0.82	–	35
C25	0.31	0	45	84
	0.32	0.8	–	16
C50	0.31	0	47	84
	0.33	0.82	–	16
C75	0.31	0	47	88
	0.32	0.76	–	12
C100	0.30	0	46	100

### Magnetic anisotropy and interparticle interactions

**Figure 5:**
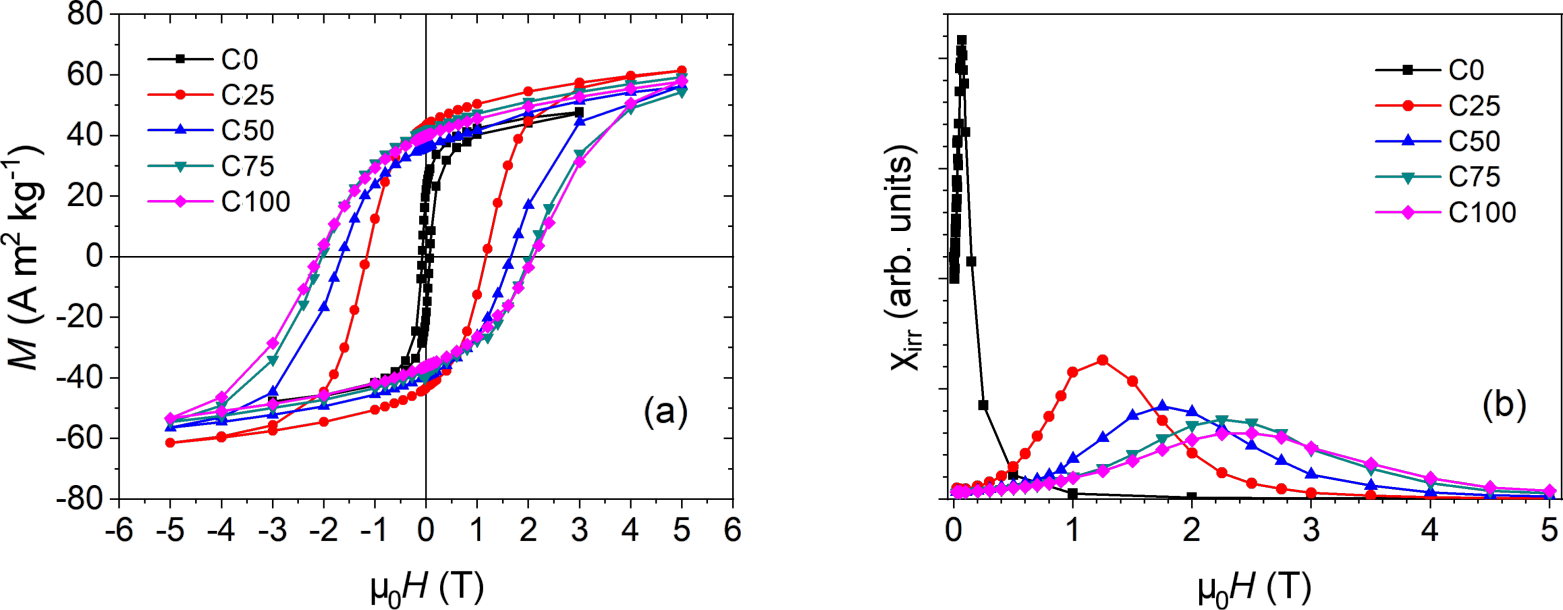
(a) Magnetization vs field curves and (b) switching field distribution measured at 5 K for sample C0 (black squares), C25 (red circles), C50 (blue triangles), C75 (dark cyan reversed triangles), and C100 (magenta diamonds).

The evolution of the magnetic anisotropy with respect to the cobalt content can be clearly observed from *M*(*H*) curves measured at 5 K (Figure 5a and Table 3). The *M*(*H*) loops show a progressive increment in coercivity with increasing Co content. We can roughly estimate the anisotropy constant assuming a Stoner–Wohlfarth model, neglecting for the moment the presence of interparticle interactions, and considering the samples as ensembles of randomly oriented NPs with uniaxial anisotropy, given that their reduced remanence is quite close to 0.5. Thus the anisotropy constant can be deduced from *K = H*_K_*M*_S_/2, where the anisotropy field is *H*_K_ = *H*_C_/0.48 [12,46]. The values of *K* reproduce the coercivity trend with respect to the Co content – the same trend shown by the remanent magnetization, too. The saturation magnetization increases with the introduction of Co with respect to the pure Mn-ferrite, then its value remains constant for all the Co-doped samples, within the experimental error (Table 3). Since the spin of Co^2+^ (3 µ_B_) is smaller than Mn^2+^ (5 µ_B_) and Mn^3+^ (4 µ_B_), a decrease in magnetization should be expected with increasing cobalt content. On the other hand, a large variation in the magnetization can be connected to different populations of Co^2+^ cations in octahedral and tetrahedral sites [19]. The *M*_R_/*M*_S_ ratio shows a step increment with the substitution of Co in place of Mn, changing the value from about ≈0.4 to ≈0.6, where it then remains constant for all Co-doped samples, suggesting that the samples have uniaxial anisotropy. While bulk CoFe_2_O_4_ has an ideal cubic magnetic anisotropy, the finite size effects on nanoparticles can suppress such behaviour showing only a small tendency to the cubic symmetry [2,12,19].

**Table 3:** Remanent magnetization (*M*_R_), saturation magnetization (*M*_S_), reduced remanence (*M*_R_/*M*_S_) and coercive field obtained from *M*(*H*) curves (μ_0_*H*_C_) calculated for each sample from *M*(*H*) loops and the average one from the SFD curves (μ_0_*H*_C_^SFD^). The intensity of Δ*M* plots is provided as a measure of the interaction intensity to be compared with the average dipolar energy trend (*E*_dip_). All curves were measured at 5 K. Uncertainties in the last digit are given in parentheses.

Sample	*M*_R_ (A m^2^ kg^−1^)	*M*_S_ (A m^2^ kg^−1^)	*M*_R_/*M*_S_	μ_0_*H*_C_ (T)	*K* (kJ m^−3^)	μ_0_*H*_C_^SFD^ (T)	Intensity Δ*M* plot (arb. units)	*E*_dip_ (J)

C0	21(2)	51(2)	0.42(5)	0.074(1)	20(1)	0.073(1)	−0.764(5)	9.78(5) × 10^−20^
C25	43(2)	67(2)	0.64(5)	1.16(1)	405(5)	1.23(1)	−0.335(5)	1.69(5) × 10^−19^
C50	38(2)	66(2)	0.58(5)	1.66(1)	570(5)	1.76(1)	−0.323(5)	1.64(5) × 10^−19^
C75	40(2)	68(2)	0.59(5)	2.03(1)	720(5)	2.26(1)	−0.321(5)	1.74(5) × 10^−19^
C100	38(2)	68(2)	0.56(5)	2.10(1)	744(5)	2.43(1)	−0.297(5)	1.74(5) × 10^−19^

The DCD protocol provided additional information about the magnetization reversal of each sample. The derivative of *M*_DCD_ with respect to the reversal field, χ_irr_ = d*M*_DCD_/dµ_0_*H*, represents the irreversible component of the susceptibility. This quantity is generally defined as the switching field distribution (SFD) [47–50], being directly proportional to the energy barrier distribution, which produces a distribution of coercivities in the nanoparticle ensemble. As we can observe in Figure 5b, the average field of the SFD curve changes to higher values with respect to the Co content, reflecting the anisotropy increment due to Co (Table 3). Moreover, we can evidence that the SFD becomes broader with increasing Co, as if the Co distribution is not homogenous through the particles, thus producing larger variability in the magnetic anisotropy.

We investigated the nature and strength of interparticle interactions in the samples by means of the so-called Δ*M* plot. The DCD and IRM remanent magnetization are related by the so-called Wohlfarth equation [51] that Kelly et al. rewrote as [32]:

[7] $$\Delta M=M_{\text{DCD}}\left(H\right)-1+2M_{\text{IRM}}\left(H\right)$$

**Figure 6:**
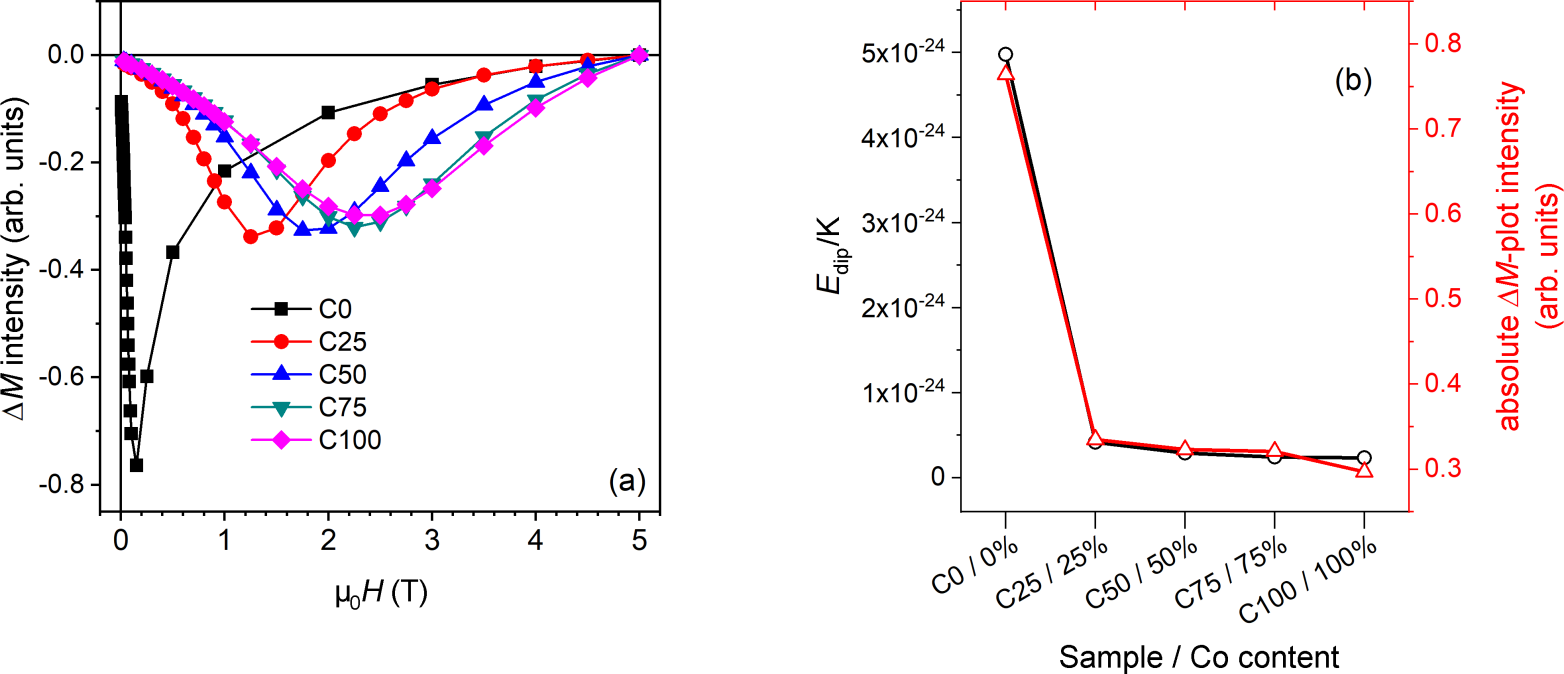
(a) Δ*M* plot curves measured at 5 K for sample C0 (black squares), C25 (red circles), C50 (blue triangles), C75 (dark cyan reversed triangles), and C100 (magenta diamonds). (b) The absolute intensity of interactions obtained from the Δ*M* plot is shown with respect to Co content (red triangles). The trend is the same exhibited by the dipolar coupling energy scaled by the effective anisotropy of each sample (black circles).

For an ensemble of non-interacting magnetic nanoparticles with uniaxial anisotropy, this curve describes a straight line. On the other hand, negative Δ*M* values are usually observed in the case of the prevalence of demagnetizing (e.g., dipole–dipole) interactions; positive values are attributed to interactions promoting the magnetized state (e.g., direct exchange interactions). As suggested by the thermal independent behavior of the FC curves at low temperature, all samples exhibit a marked interaction regime, with negative deviations connected to prevalent dipole-dipole interactions (Figure 6a). This is expected for bare particles in close contact but without any major coalescence [52]. The larger intensity of the interactions belongs to C0, and decreases with respect to the Co content with an exponential decay. We roughly estimated the dipolar interaction energy as [53]:

[8] $$E_{\text{dip}}\approx \frac{\mu_{0}}{4\pi }\frac{\mu^{2}}{d^{3}}$$

where µ is the magnetic moment of the single particle and *d* the distance between particle centers (considered as point dipole), calculated as the average particle diameter, assuming that the samples consist of bare particles in direct contact (Table 3). The average dipolar energy shows an increasing trend with respect to the amount of Co, which is at odds with the trend shown by the intensity of the Δ*M* plots [49]. The evaluation of the dipolar energy does not take into account the different anisotropy contribution of each sample. Indeed, if we normalize the dipolar energy by the estimated anisotropy constant, we can perfectly reproduce the interaction trends depicted by the Δ*M* plot (Figure 6b). These conclusions suggest that the higher anisotropy of single particles produced by the Co doping reduces the effective coupling. When the anisotropy is small, the interactions can exert the strongest effect. This leads to the spinglass-like interacting regime observed in C0, owning this sample the smallest anisotropy.

These results illustrate that for ensembles of interacting particles, higher individual NP energy barriers work against the collective interaction behavior, which is in agreement with the observation of our recent work [54]. This is a crucial aspect for the implementation of such systems in applications and, in this framework, our synthesis method, despite its simplicity, allows engineering of the overall magnetic behaviour of the ensembles that is determined primarily by their magnetic anisotropy and the interparticle interaction regime. Those two aspects can be effectively modulated by controlling the Co content without sacrificing control over particle size. Nevertheless, our study evidences that a significant change in the magnetic properties occurs in the concentration range between 0 and 25% of cobalt, while subtler variations occur due to incremental additions of Co in substitution of Mn.

## Conclusion

The effect of cobalt doping on the magnetic properties of Mn_1−_*_x_*Co*_x_*Fe_2_O_4_ nanoparticles prepared by low-energy ball milling was investigated. Small effects are observed regarding the structure of the sample, while the average particle size and shape remain almost constant. All samples systematically show a lattice parameter smaller than in bulk, independently of the Co content. On the other hand, the magnetic properties change remarkably upon Co-doping. The main effect is related to the magnetic anisotropy, which increases sharply with the substitution of Mn by 25% Co, and then more gradually with further additions of Co. Moreover, the effect of Co is evident in the reduced remanence, which shows values typical for uniaxial symmetry. In addition, Co substitution has a profound effect on the interparticle interactions. The Δ*M* plots evidence a dipolar-based interaction regime for all samples, but the intensity of the interactions is mitigated by the single particle anisotropy. This play between the interparticle interactions and the single particle anisotropy becomes clear when analysing the trend shown by the samples. C0 owns the weakest dipolar interactions among the samples. Nevertheless, having also the smallest anisotropy, it exhibits the strongest effective interaction regime, actually showing a super-spin-glass behavior. Tuning the anisotropy is one way to control the overall magnitude of the interactions, opening new interesting perspectives for controlling the magnetization reversal of concentrated NP systems for specific applications.

## Supporting Information

An example of a typical X-ray diffraction pattern of the amorphous phase obtained immediately after the milling process for sample C0 (Figure S1). ZFC and FC curves measured for all samples are shown in Figure S2. For sample C0, the AC susceptibility vs temperature curves measured at different frequencies are shown in Figure S3. Figure S4 presents the fits of the frequency dependence of the maximum of the curves (*T*_P_) using the Arrhenius model, the Vogel–Fulcher law and the power law. Only the last two models provide physically meaningful parameters and are reported in Table S1.

File 1 Additional experimental results.
