# Supplementary material for: Co-doped MnFe2O4 nanoparticles: magnetic anisotropy and interparticle interactions
Source: Beilstein J Nanotechnol. 2019 Apr 12;10:856–65. doi: 10.3762/bjnano.10.86 (PMC6466680; doi:10.3762/bjnano.10.86)
Supplement: File 1 — Additional experimental results. [file Beilstein_J_Nanotechnol-10-856-s001.pdf]

BEILSTEIN JOURNAL OF NANOTECHNOLOGY

Supporting Information

for

Co-doped $MnFe_2O_4$ nanoparticles: magnetic anisotropy and interparticle interactions

Bagher Aslibeiki, Parviz Kameli, Hadi Salamati, Giorgio Concas,
Maria Salvador Fernandez, Alessandro Talone, Giuseppe Muscas and Davide Peddis

Beilstein J. Nanotechnol. **2019,** *10,* 856–865. doi:10.3762/bjnano.10.86

Additional experimental results

License and Terms: This is a supporting information file under the terms of the Creative Commons Attribution License (http://creativecommons.org/licenses/by/4.0). Please note that the reuse, redistribution and reproduction in particular requires that the authors and source are credited.
The license is subject to the *Beilstein Journal of Nanotechnology* terms and conditions: (https://www.beilstein-journals.org/bjnano)

Figure S1

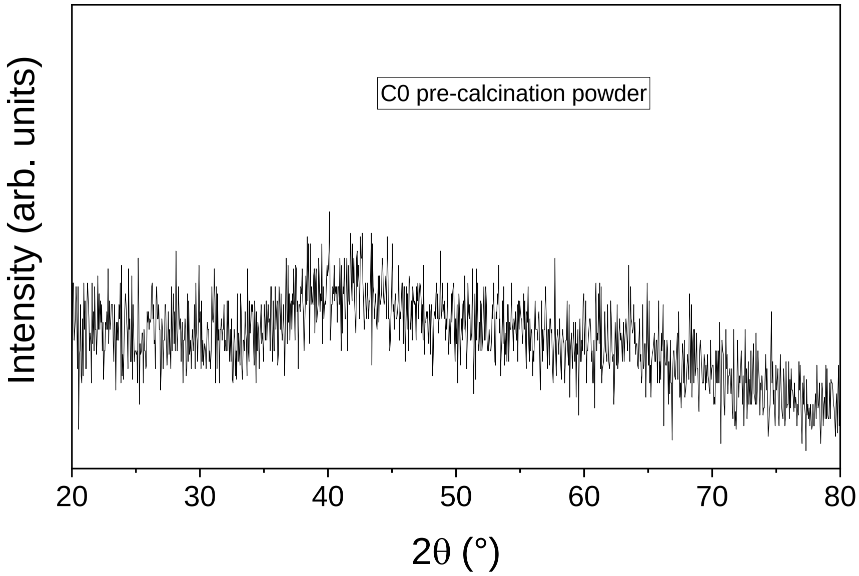

Figure S1: The XRD pattern of sample C0 immediately after the milling process is reported as an example. All samples consist of an amorphous powder at this stage of the synthesis.

Figure S2

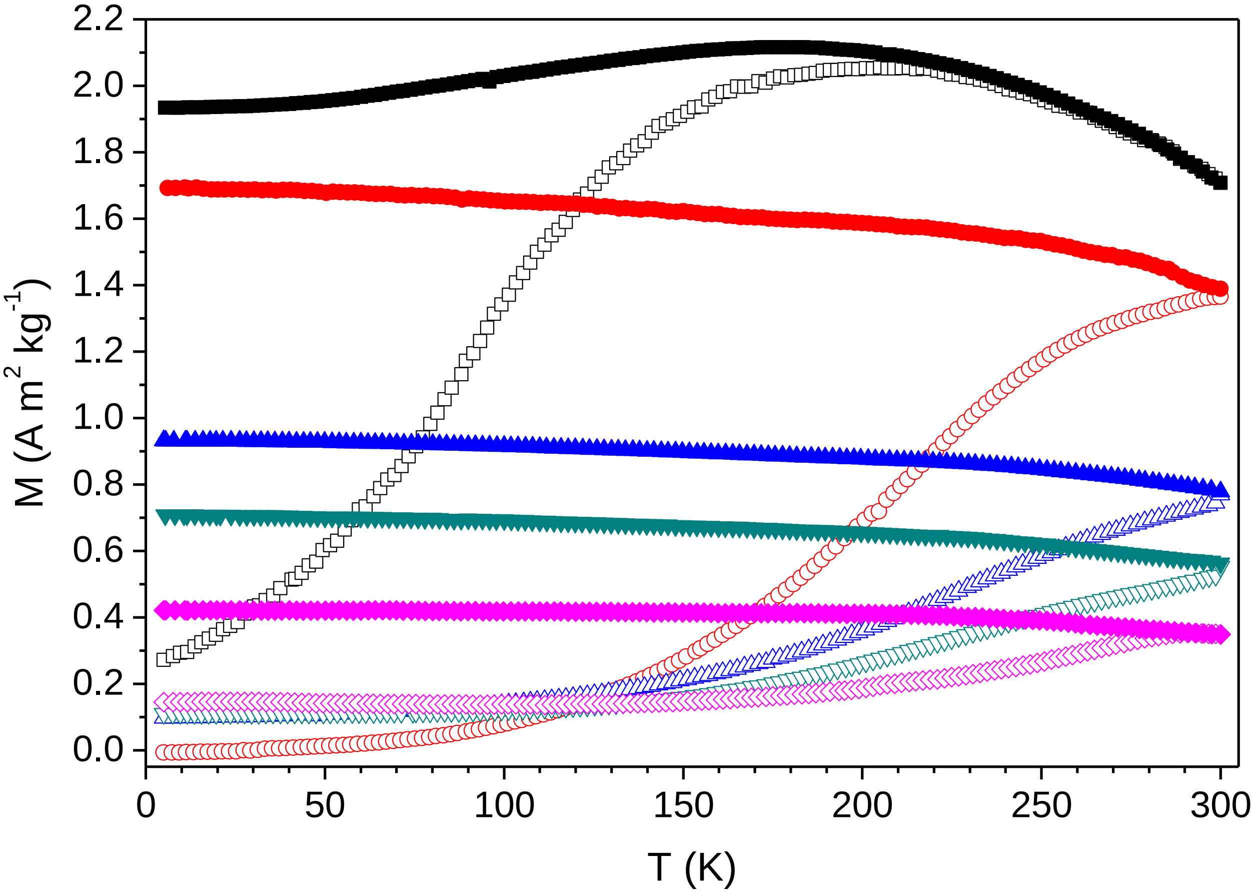

Figure S2: ZFC (empty symbols) and FC (full symbols) for sample C0 (black squares), C25 (red circles), C50 blue triangles), C75 (dark cyan reversed triangles), and C100 (magenta diamonds).

Figure S3

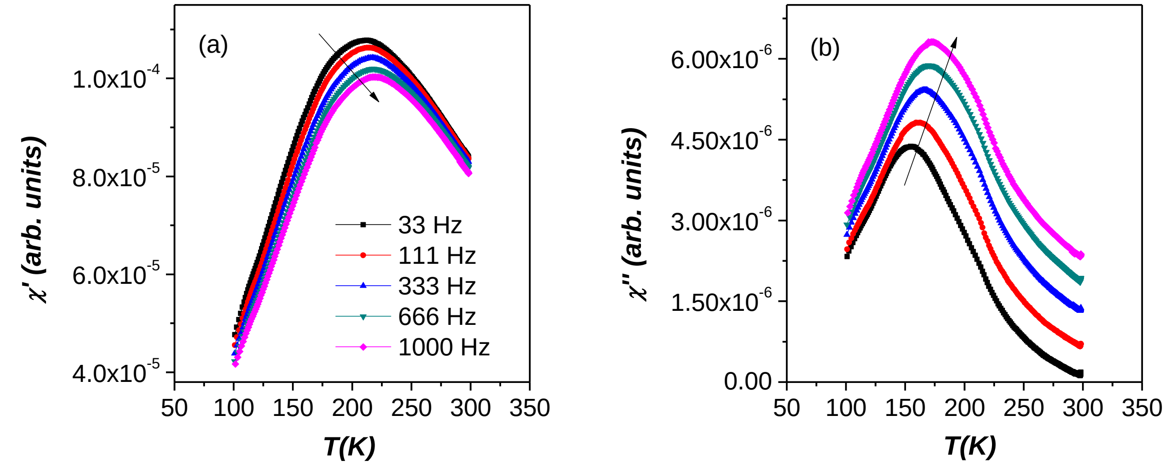

Figure S3: AC susceptibility vs temperature measured at different frequencies for sample C0. Panel (a) in-phase component; panel (b) out-of-phase component.

Figure S4

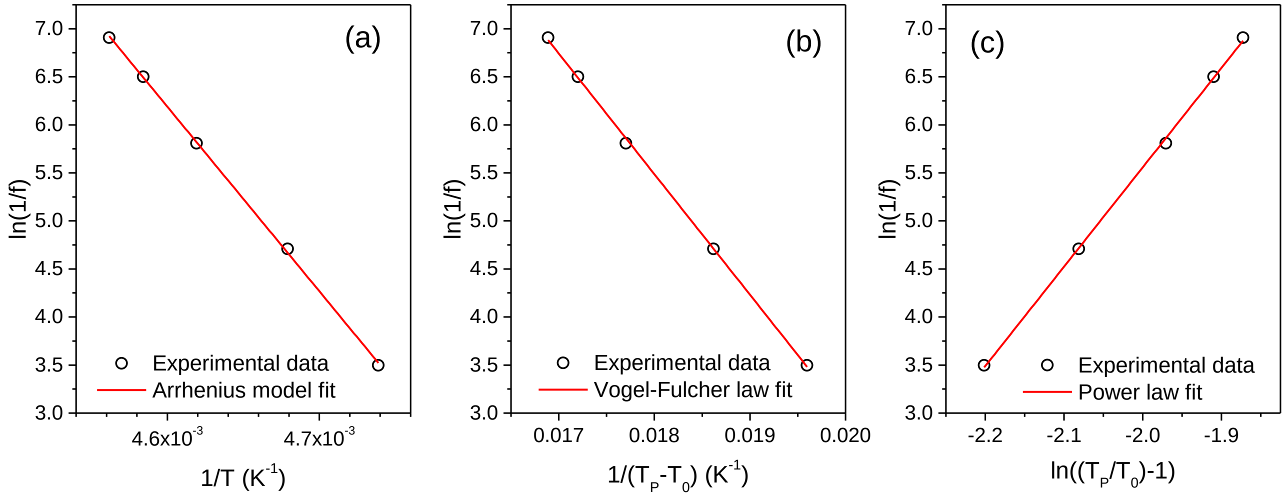

Figure S4: Frequency dependence of T_P in AC curves of sample C0 fitted with the Arrhenius model (a), the Vogel–Fulcher law (b) and the Power law (c).

Table S1

Table S1: Parameter obtained from the AC data fits of sample C0 using the Vogel–Fulcher and the power law.

Model	Values	
Vogel–Fulcher	τ_0 (s)	7(2)×10^{-13}
	E/k$_B$ (K)	1250(20)
	T_0 (K)	160(3)
Power law	τ_0 (s)	4(1)×10^{-12}
	zv	10.3(2)
	T_g (K)	190(3)
